# Modeling fisheries and carbon sequestration ecosystem services under deep uncertainty in the ocean twilight zone

**DOI:** 10.1007/s13280-024-02044-1

**Published:** 2024-08-29

**Authors:** Maartje Oostdijk, Laura G. Elsler, Julie Van Deelen, Willem L. Auping, Jan Kwakkel, Amanda Schadeberg, Berthe M. J. Vastenhoud, Claudiu Eduard Nedelciu, Fabio Berzaghi, Raul Prellezo, Mary S. Wisz

**Affiliations:** 1 Faculty of Agricultural Sciences, Agricultural University of Iceland, Keldnaholt, Árleynir 22, 112 Reykjavík, Iceland; 2 Ocean Sustainability, Governance and Management, World Maritime University, Fiskehamnsgatan 1, 211 18 Malmö, Sweden; 3 Science Institute, University of Iceland, Saemundargata 2, 101 Reykjavik, Iceland; 4 Harvard. T.H. Chan School of Public Health, Boston, MA 02115 USA; 5 Policy Analysis Section, Department of Multi-Actor Systems, Faculty of Technology, Policy and Management, Delft University of Technology, Jaffalaan 5, 2628 BX Delft, The Netherlands; 6 Environmental Economics and Natural Resources Group, Wageningen University, Hollandseweg 1, 6706 KN Wageningen, The Netherlands; 7 Environmental Policy Group, Wageningen University, Hollandseweg 1, 6706 KN Wageningen, The Netherlands; 8 National Institute of Aquatic Resources, Technical University of Denmark, Kemitorvet 201, 2800 Kgs. Lyngby, Denmark; 9 Department of Geography, System Dynamics Group, University of Bergen, Fosswinckelsgate 6, 5007 Bergen, Norway; 10 AZTI. Marine Research Unit. Txatxarramendi Ugartea Z/G, 48395 Txatxarramendi, Sukarrieta, Spain

**Keywords:** Blue carbon, Carbon sequestration, Deep uncertainty, Mesopelagic fishery, Social-ecological modeling

## Abstract

Mesopelagic fishes are a vital component of the biological carbon pump and are, to date, largely unexploited. In recent years, there has been an increased interest in harvesting the mesopelagic zone to produce fish feed for aquaculture. However, great uncertainties exist in how the mesopelagic zone interacts with the climate and food webs, presenting a dilemma for policy. Here, we investigate the consequences of potential policies relating to mesopelagic harvest quotas with a dynamic social-ecological modeling approach, combining system dynamics and global sensitivity analyses informed by participatory modeling. Our analyses reveal that, in simulations of mesopelagic fishing scenarios, uncertainties about mesopelagic fish population dynamics have the most pronounced influence on potential outcomes. The analysis also shows that prioritizing the development of the fishing industry over environmental protection would lead to a significantly higher social cost of climate change to society. Given the large uncertainties and the potential large impacts on oceanic carbon sequestration, a precautionary approach to developing mesopelagic fisheries is warranted.

**Supplementary Information:**

The online version contains supplementary material available at 10.1007/s13280-024-02044-1.

## Introduction

As global demands for food and goods rise (Pace and Gephart 2017; Hickel et al. 2022), affluent regions are leaving a noticeable environmental footprint (Chancel 2022). In parallel, biodiversity is declining (Bjelle et al. 2021; Pörtner et al. 2023), further complicated by the accelerating impacts of climate change (Navarro-Racines et al. 2020), affecting wildlife and ecosystems (Pörtner et al. 2023).

These challenges are intertwined: climate change exacerbates biodiversity loss (Pörtner et al. 2023), while ecosystems absorb atmospheric carbon (Boyd et al. 2019; Pörtner et al. 2023). As the global population grows, there is an increased demand for animal protein (Naylor et al. 2021). Responding to this demand, aquaculture production has risen, driven by the need to address depleting wild fish stocks (FAO 2022). However, this growth in aquaculture raises a dilemma: aquaculture fish require protein, often sourced from wild-caught forage fish (Froehlich et al. 2018).

Balancing the ecosystem impacts of forage fish harvesting against the food provided by aquaculture requires careful consideration. This decision-making process is complex, involving high stakes and significant uncertainties (Marchau et al. 2019). To address these complexities, transdisciplinary approaches with active stakeholder involvement are crucial (Bernstein 2015).

There is limited possibility for expanding marine capture fisheries to address the growing demand for seafood (Free et al. 2022). In recent decades aquaculture production has grown steeply, with a lot of growth in blue foods related to aquaculture growth (Naylor et al. 2021). While some aquaculture is supported by land-based production (e.g., soy), and trophic levels of piscivorous aquaculture fish (e.g., salmon) have decreased in recent years (Cottrell et al. 2021), aquaculture production of piscivorous species still relies on fishmeal supplied by wild capture of forage fishes. Furthermore, climate change is decreasing viable options for expanding blue food production (Free et al. 2022).

A large and (almost) unexploited marine ecosystem is the mesopelagic zone, a zone in the open ocean 200–1000 m deep. It is too dark for photosynthesis but receives sufficient light for visibility (Robinson et al. 2010) and is also called the ocean twilight zone. The mesopelagic ecosystem provides a diversity of regulating (i.e., carbon and nutrient cycling), and supporting (i.e., prey species for important commercial and protected species) ecosystem services (St. John et al. 2016; Iglesias et al. 2023). Harvesting the mesopelagic zone promises large seafood production volumes for aquaculture input (Alvheim et al. 2020). The viability of this fishery, however, remains uncertain. Biomass estimates have been high, but also highly variable (Hidalgo and Browman 2019), ranging from 1.8 to 16 Gt (25–75% quartile ranges; Proud et al. 2019). An assess

Mesopelagic fish exploitation may be economically viable from the fishing operation perspective (Prellezo 2019; Paoletti et al. 2021). However, this is still uncertain and contingent on technicalities such as processing abilities and catchability. Mesopelagic fish have a low catchability due to their widespread and patchy distribution (Olivar et al. 2012; Proud et al. 2017), their effective trawl avoidance (Kaartvedt et al. 2012), and variability in spatial patterns of occurrence (Olivar and Beckley 2022). The fishery will likely have high operating costs due to the large amounts of fuel needed, in addition to investment in new processing methods (Paoletti et al. 2021). Due to their high-fat content, mesopelagic fish deteriorate quickly upon harvesting, requiring the likely development of specialized onboard processing equipment (Paoletti et al. 2021). However, current and upcoming effort limitations on current fisheries, growing fish and aquaculture markets, and technological innovation may make mesopelagic fishing more profitable in the future (Prellezo 2019).

Large-scale fishing in the mesopelagic zone could provoke a trade-off between seafood production and other vital ecological functions of mesopelagic fish (St. John et al. 2016). Several mesopelagic fish and zooplankton species migrate vertically (Passow and Carlson 2012; Davison et al. 2013), feeding at the surface at night and hiding from predators at depth during the day. During vertical migration, fish transport carbon from the surface to the deep sea, where carbon is stored for longer periods of time, 100 years, and longer if excreted at depths > 1000 m (Passow and Carlson 2012). With their large biomass and this collective behavior, mesopelagic species contribute to carbon sequestration in the ocean (Martin et al. 2021; Saba et al. 2021) at a scale that may be globally significant (estimated 41% of total active carbon export), but it is highly uncertain (0.9–3.6 Pg yr^−1^; Boyd et al. 2019). The cost to society associated with reductions in carbon sequestration is likely to be high but uncertain (Barange et al. 2017; Jin et al. 2020).

The history of fisheries tells a cautionary tale about the importance of governance in maintaining ecosystem services. Generally, fish populations and marine ecosystems are in better condition in fisheries with more sophisticated management regimes (Melnychuk et al. 2017). At present, there is little management of the mesopelagic zone (Schadeberg et al. 2023), with a few exceptions, such as a precautionary moratorium on the US West coast (Dowd et al. 2022) and a precautionary catch-based limit in Iceland (Marine Research Institute 2015). New fisheries, like straddling and highly migratory fish stocks governed by the UN Fish Stocks Agreement (UNFSA 1995), will require cautious conservation measures per Article 6(6), overseen by regional fisheries management organizations (RFMOs). Despite the minimal investigation into RFMO implementation (Caddell 2018), their effectiveness varies (Cullis-Suzuki and Pauly 2010). Lobby groups influence ecosystem-based fisheries management, potentially impacting mesopelagic fisheries (Orach et al., 2017; Oostdijk et al. 2022). The impact of decision-making uncertainties, including lobby group influence on RFMO decisions regarding mesopelagic fisheries, remains unquantified.

Sustainability decision-making is often described as a “wicked problem,” in which facts are deeply uncertain, stakes are high, values are in dispute, and decisions are urgent (Funtowicz and Ravetz 1990). Sustainability decisions invariably involve values, leading to calls for transdisciplinary approaches to decide on desirable outcomes and outcomes to avoid (Brown et al. 2010). Consequently, analytical approaches have been established that combine system dynamic modeling, advanced sensitivity analysis, and participatory approaches to weigh the impact of different decisions on the outcomes at stake (Kwakkel et al. 2016). These approaches can, for instance, be used to discover “worst-case scenarios” for outcomes that are of interest, which is useful for decision-makers who may want to govern using the precautionary approach. The precautionary approach applies tactics that try to avoid those worst outcomes, for instance, by implementing a low harvest limit based on the lower bound estimates of stock size, as is implemented by the International Council for Exploration of the Seas (ICES) (Lassen et al. 2014) to avoid stock collapse and adverse ecosystem impacts. In this article, we apply a system dynamics model combined with advanced sensitivity analyses to investigate governance scenarios for harvesting mesopelagic fish, and we weigh the profit of harvest against the societal cost of increased exposure to damage from climate change. We also identify the key uncertainties in the outcomes in catches, biomass of the population and carbon sequestration and the social cost of the loss of carbon sequestration ecosystem services. We follow the approach presented by Moallemi et al. (2020) to explore outcomes informed by stakeholder participation and model parameter uncertainties. The combination of these approaches has been used in fields such as water resource management, climate adaptation, public health, national defense and security, and energy policy (Marchau et al. 2019). To our knowledge, this is the first application of such approaches (i.e., the combined use of SD with deep uncertainty analyses) to an ocean sustainability challenge.

## Materials and methods

The methods are centered around (1) the construction of a system dynamics model (Forrester 1961), and (2) using Decision-Making Under Deep Uncertainty (DMDU) methods (i.e., elaborate sensitivity analysis on possible model outcomes) to arrive at possible robust decisions under deep uncertainty. Stakeholder participation is used to inform the system dynamics model and decisions/outcomes to focus on in the DMDU method. See Fig. S1 for an overview of the methods we used.

### The system dynamics model

System dynamics uses coupled equations and is well suited to study complex social-ecological systems (Martin and Schlüter 2015). System dynamics has been used before in modeling fisheries to explore participatory scenarios for small-scale fisheries, or to study patterns of overexploitation in existing industrial fisheries (Röckmann et al. 2012; Perissi et al. 2017; Pouso et al. 2019).

We constructed a stylized global model, which is not spatial and is based on coupled difference equations. This model contains a simplified set of equations representing real-world dynamics (Lade et al. 2019; Eppinga et al. 2023).

**Fig. 1:**
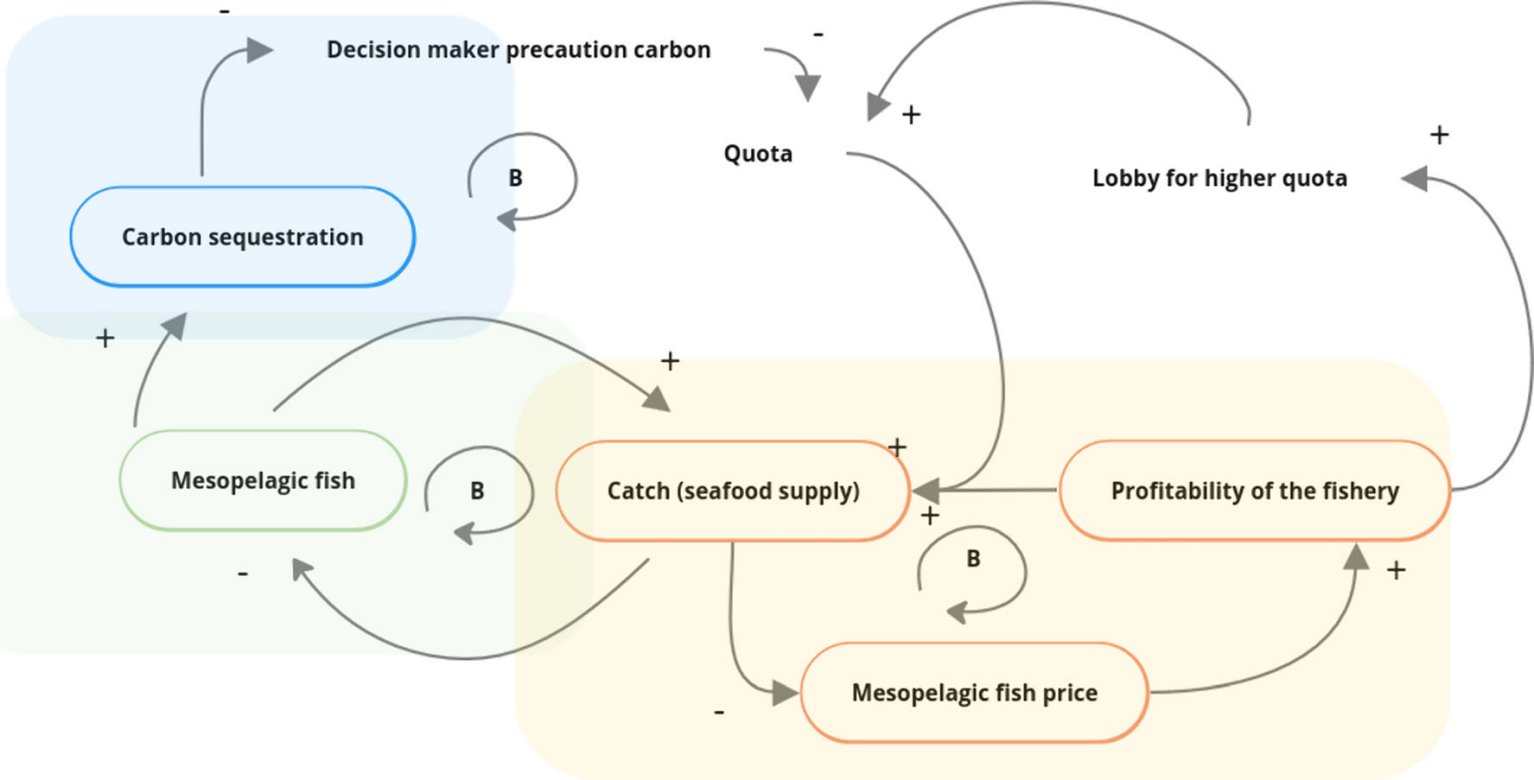
Causal loop diagram of the core structure of the model, + signs show a positive relationship between model variables, and − signs show a negative relationship between model variables. Feedback loops are indicated with a circular arrow. All feedback loops are balancing (B). See model formulas and Table 1 (bio-economic parameters) and 2 (governance parameters) for more explanation regarding the modeled variables. (green color represents population dynamic parameters, blue represents carbon cycle parameters, red represents economic parameters, while white represent governance parameters)

The model consists of four main modules and is a reworked version of the model used by Van Deelen (2021). The first module models mesopelagic fish dynamics (see main causal dynamics for the modules in Fig. 1), the second module models the oceanic carbon cycle component, which models key attributes of the ocean biological pump and the role of mesopelagic fish therein. The third module models fisheries economics and food provision components, including the economic decisions to fish, and their relationship to profitability and efficiency. The fourth module is the governance component, which models the way quota setting is impacted by different economic actors.

We used participatory methods to inform the structure and scenarios in the system dynamic model, which are detailed in Appendix S1. Briefly, we relied on a previous interview campaign with experts (n = 20) (Oostdijk et al. 2022) and a workshop largely focused on extreme outcomes, in which we combined a (pre-workshop) survey and participatory modeling (Kraan et al. 2022). The pre-workshop survey showed that participants were interested in several main themes or (extreme) outcomes (e.g., the impact of fishing on the status of mesopelagic fish populations, especially given the lack of detailed knowledge about these populations, the amount of achievable harvest from the standpoint of food security and the key role of the mesopelagic species in the ecosystem and carbon cycle, Appendix S1). We integrated several of these concerns into the structure of the SD model, to achieve a model that was able to address these outcomes of interest. Moreover, during participatory modeling sessions during the actual workshop we addressed causal connections that could lead to outcomes of interest. These resulted in quite complicated system maps with many causal links (Fig. S2–S4), which the author team collaboratively summarized into key drivers, that were validated by literature review.

The system dynamics model was implemented in Python. The outcomes of the model are the range of possible outcomes for mesopelagic biomass, carbon sequestration (with and without fishing), harvest levels, fishery profits and the cost of fishing in terms of the computed social cost of carbon.

### Model structure and main equations

*Mesopelagic biomass, fishing, costs, and profits:* We constructed a simplified social-ecological model departing from a Gordon-Schaefer surplus production model for mesopelagic fish (Schaefer 1957):

1 $$M_{t + 1} = M_{t} + M_{t} *r(1 - M_{t} /K) - H_{t}$$

*M* is the size of the mesopelagic fish population, for simplicity’s sake this is modeled as a single biomass pool. *r* is the relative growth rate of mesopelagic fish, which depends on the amount of mesopelagic fish with respect to the carrying capacity *K*. *H* is harvest, which is modeled as follows:

2 $$H_{t} = qE_{t} *M_{t}$$

where Harvest *H* is proportional to effort times catchability *q*, effort *E* and the size of the mesopelagic stock *M* (Schaefer 1957).

Fishing effort is proportional to the profitability of the fishery and is modeled as follows, adapted from (Fryxell et al. 2017):

3 $$E_{t + 1} = E_{t} + ({\;{alpha}}[p_{t} (H_{t} ) - cE_{t} ]),$$

With the constraint being that effort does not increase if *H*_*t*_ >/= to quota (*Q*). We assume that the effort of the previous year impacts the current year's effort. alpha is a factor that modulates the change in effort contingent on revenue and cost, assuming *sp* is the price obtained for mesopelagic fish and *c* is the cost of fishing (Fryxell et al. 2017). Effort has a near zero initial value as the structure of Eq. 3 does not allow for the fishery to start off from zero.

Profitability (*π*) is defined as the sale price (*p*) of mesopelagic fish minus the cost of fishing (*c*):

4 $$\pi_{t} = p_{t} H_{t} - cE_{t}$$

Sale price (*p*) of mesopelagic fish is determined endogenously based on the size of the harvest, equation adapted from Elsler et al. (2021), Fryxell et al. (2017):

5 $$p_{t} = \, X*{\;{gamma}}*H_{t} \left( { - 1*{\;{Beta}}} \right)$$

where gamma is the initial price of mesopelagic fish, and Beta is the parameter that adjusts the price when demand is increased. *X* is a modifier for demand, representing the trend of increasing demand for fishmeal/fish oil with increasing aquaculture production and no or little shift to land-based feeds (Froehlich et al. 2018).

*Carbon dynamics:* We expanded the model to include a carbon and governance component. Total carbon injected is based on cumulative carbon injected by weight of mesopelagic fish, and the percentage that is injected through respiration, fecal pellets, and the mortality pathway are modeled as follows:

6 $$C_{i,t + 1} = M_{t} \mu f_{i} + C_{i,t} \left( {1 - \left( {\frac{1}{{s_{i} }}} \right)} \right);\quad {\text{Where}}\;\;i = \left\{ {r,f,m} \right\}$$

where $\mu$ is the carbon injected per year per weight of mesopelagic fish, *f*_*r*_*, f*_*f*_ and *f*_*m*_ are fractions of carbon injected through respiration, fecal and mortality pathways, respectively. *C*_*r*_, *C*_*f*_*,* and *C*_*m*_ are carbon injected through respiration, fecal, and mortality pathways, respectively. *s*_*r*_*, s*_*f*_, and* s*_*m*_ represent the duration of sequestration of each of the pathways.

Total carbon sequestered due to vertically migrating fish is modeled as follows:

7 $$C_{it} = C_{r,t} + C_{f,t} + C_{m,t}$$

*Governance:* Quota setting is impacted by the amount of carbon sequestered by mesopelagic fish and the fishery profitability. P This effect takes place through two parameters: one for the impact of fishing industry lobby and one for the impact of government environmental concern:

8 $$Q_{t} = Q\_0*{\;{ FL}}_{t} * \, E_{t}$$

where *Q*_0 is the initial level of quota suggested by a fictive advisory organ, FL is the effect of the fishing industry lobby on that quota, and *E* is the effect of the government level of environmental concern due to loss of carbon sequestration function.

The lobby effect is impacted by profitability once the profitability crosses a threshold, based on profitability in other fisheries (i.e., if the fishery becomes equally or more profitable than current fisheries for small pelagic species, this effect will occur):

9 $$\left\{ {\begin{array}{*{20}l} {{\;{FL}}_{t} = {\;{FL}}\_{\;{effect}}} \hfill & {{\text{if}}\;\;\pi_{t} /\left( {cE_{t} } \right) > pl} \hfill \\\ 1 \hfill & {{\text{otherwise}}} \hfill \\ \end{array} } \right.$$

where FL_effect is the predetermined effect size of fishing lobby on quota setting, and *pl* is the profit level that should be crossed for the fishery to become commercially interesting enough for fishing lobby to take place.

The environmental concern effect also comes into play once carbon sequestration loss crosses a certain threshold compared to carbon sequestration without fishing.

10 $$ \left\{ {\begin{array}{*{20}l} {{{E}}_{t} = E\_{ef\!fect}} \hfill & {{\text{if}}\;\;C_{it} < el \, *C_{it = 5}} \hfill \\ 1 \hfill & {{\text{otherwise}}} \hfill \\ \end{array} } \right. $$

where *E*_effect is the predetermined effect size of environmental concern on quota setting, which comes into play if total yearly carbon sequestration is below a conversion factor (*el*) times its level in the initial phase of the simulation, virtually without fishing.

The monetised climate impact (using estimates of the social cost of carbon) of mesopelagic fishing is defined as the difference in total sequestered carbon between scenarios with and without fishing, multiplying this by the social cost of carbon. The cost to society of harvesting mesopelagic fish is measured in lost sequestration potential compared to a pristine population times a CO_2_ conversion coefficient and times the social cost of carbon per tonne CO_2_.

We ran the model for a simulation of 50 years with a yearly time step. Table 1 details the model parametrization. Some additional background on parametrization can be found in Appendix S2, and Table 2 presents the governance scenarios.

**Table 1:** System dynamic model parametrization (green color represents population dynamic parameters, blue represents carbon cycle parameters, and red represents economic parameters). Upper and lower bounds of confidence intervals were generally ± 75%, unless literature indicated differently (e.g., recent studies regarding mesopelagic biomass do not indicate biomass values higher than 4.5 Gt). Several theoretical parameters (e.g., alpha) have purposefully wide ranges

Module	Variable	Parameter value	Units	Reference	Range for deep uncertainty analysis
Population dynamics	Initial Mesopelagic fish biomass (*M*_0)	3	Gt	Slightly higher than Anderson et al. (2019), as range in Proud et al. (2019) heavy right skewed (uncertainty range: Hidalgo and Brownman 2019; Proud et al. 2019; Irigoien et al. 2014). Dornan et al. (2022) find that estimated biomass of lanternfish were 1.8 and 3.8 times greater than previous net-based biomass estimates, combining acoustic and survey approaches for the southern ocean. Which is in line with Andersson et al. (2019) findings	1.5–4.5
	Carrying capacity (K)	3	Gt	Slightly higher than Anderson et al. (2019), as range in Proud et al. (2019) is heavily right skewed	1.5–4.5
	Growth rate (*r*)	0.9	1/Yr	Thorston et al. (2017), generation doubling time around 1.4–4.4 years (Froese et al. 2017)	0.225–1.575
Carbon	Conversion bodyweight mesopelagic fish to injected carbon ($\mu$)	0.77	Dmnl	Davison et al. (2013)	^a^
	% fish carbon injected through pathway	0.35 for fecal, 0.32 for respiration, 0.33 for mortality	Dmnl	Davison et al. (2013)	^a^
	Sequestration length carbon injected mesopelagic fish	103 Yr for respiration, 599 Yr for fecal pellets, 851 for deadfall	Year	Pinti et al. (2023)	25.75–180.25; 149.75–1048.25; 212.75–1489.25
	Conversion carbon to CO_2_	3.67	Dmnl	Based on the atomic mass of carbon as a fraction of CO_2_: 12/44	
Economic	Costs fishing with specialist capacity ($c$)	37,000	€/day	STECF data on pelagic seines > 40 m in EU, multiplied by 1.5 (as per Paoletti et al. 2021), see Figure C3	18.500€/- €/55,500/day
	Harvesting capacity per day (*q*)	200	Tons per unit effort (1 day at sea)	Norwegian trial fishery in Groeneveld et al. (2022)	100–300 tons per day
	Social cost of carbon (scc)	162	€/per ton CO_2_	Rennert et al. (2022) (2020 Euros)	38.6–362.28
	Alpha *a*	0.5	Dmnl	Fryxell et al. (2017)	0.1–1
	Gamma	350	Price (€//ton) when harvest = 1	(Groeneveld et al. 2022; Fryxell et al., 2017 uncertainty: Prellezo 2019, Paoletti et al. 2021)	175–525
	Price flexibility (*β*)	0.005345	Dmnl	Appendix S2, Fig. S5	0.000134–0.000935
	Demand multiplier (*X*)	1.004	Dmnl	Froehlich et al. (2018)	1–1.008

We did not do sensitivity analysis on these values as these rates are based on daily metabolic rates assumptions regarding mesopelagic fish, that are not explicitly modeled in our analysis (and relate in a non-straightforward way to growth in a surplus production model, as growth rate (r) represents both recruitment and metabolic growth), we therefore chose the baseline scenario in Davison et al. (2013) for these estimates

**Table 2:** Key management/governance parameters and uncertainties

Variable	Parameter value	Units	Reference	Range for deep uncertainty analysis
Proposed harvesting quota (*Q*_0*)*	0.3	1/Yr	ICES advice blue whiting Fmsy 0.32 (ICES 2022) (in theory this could probably be higher in case of high r, but advisory organs often take a precautionary approach with forage fish due to predation by important predatory fish (ICES 2020)	0.15–0.45
Fishing industry lobbying effect size (*FL_effect*)	Nonlinear phase shift with increased profitability 1 for profit less than *pl*, 1.2 profit above pl (*if then else statement*)	Dmnl	Scenario/assumption Fished levels or allocated total allowable catches are frequently higher than advised (e.g., Woods et al. 2015; Carpenter et al. 2016) due to industry interests. In the EU TACs were set on average 20% above advice (Carpenter et al. 2016), with the highest excess TAC being blue whiting (52%), which is a shared and migratory stock (Bjorndal and Ekerhovd 2014). This scenario of setting higher quota can also be interpreted as a scenario of IUU fishing, due to a lack of governance capacity (e.g., in the high seas) as addressed by experts in the stakeholder workshops (Appendix S2)	1–2
Profit level (*pl*) at which lobby takes place	> 20%	Dmnl	Annual Economic Report (STECF 19–06), Fig. S6	> 10- > 30%
Carbon sequestration governance effect size (*E_effect*)	Nonlinear phase shift with decreased carbon sequestration 1 for 0 loss of carbon sequestration, 0.8 for less than 50% of yearly mesopelagic carbon sequestration in year 1 of the simulation. (*if then else statement*)	Dmnl	Scenario/assumption Social norms can shift rapidly depending, sometimes accelerated by policy changes (Lenton 2020) This scenario takes into consideration that the decision maker(s) could act out of precaution. It could, for instance, be facilitated through the implementation of a carbon valuation method as addressed by experts in the stakeholder workshops (Appendix S2)	0.2–0.8
Percentage of loss of carbon sequestration at which environmental concern takes place (*el*)	50%	Dmnl	Scenario/assumption	25–75%

The biomass of all mesopelagic populations combined is very high in comparison to current commercially fished forage fish populations. Thus, we also performed a set of runs where we restricted the maximum yearly harvest to be around the size of the current annual global capture of forage fish (20 million tonnes, (FAO 2022)).

### Decision-making under deep uncertainty (DMDU) methods

DMDU methods can be used to explore structural uncertainties, such as biomass estimates or fish prices in the case of a mesopelagic fishery, and parameter uncertainties (Moallemi et al. 2020), and their consequences for decision-making. The impacts of such uncertainties and their implications for outcomes of decisions can, for instance, provide evidence warranting a precautionary approach to policy (Bisson et al. 2023). The DMDU analyses were performed in the Exploratory Modeling and Analysis (EMA) workbench 2.2 (Kwakkel 2017).

#### Experiments and uncertainty analysis

As an exploratory uncertainty analysis, we ran the model 100 000 times over the parameter space for the uncertain parameters using Latin Hypercube Sampling (Tables 1, 2). We used extra-trees feature scoring (Jaxa-Rozen and Kwakkel 2018) to select the main uncertainties that drive the model outcomes for the variables biomass, seafood supply, fishery profitability, and carbon sequestration by the migrant pump and the value of the carbon measured in the social cost of carbon.

#### Worst-case scenario discovery

The participatory methods helped us determine which outcomes we should avoid or strive for, and which are considered “worst outcomes” (Appendix S1). The worst-case scenario discovery function in the EMA workbench runs over all uncertainties (Table 1) and levers (Table 2) and filters scenarios that have overall low scores for desired outcomes (Halim et al. 2016). A worst-case scenario has, in our case, low catches, low biomass and/or low carbon sequestration.

## Results

### Exploratory analysis full system dynamic model, fishing and fisheries management

**Fig. 2:**
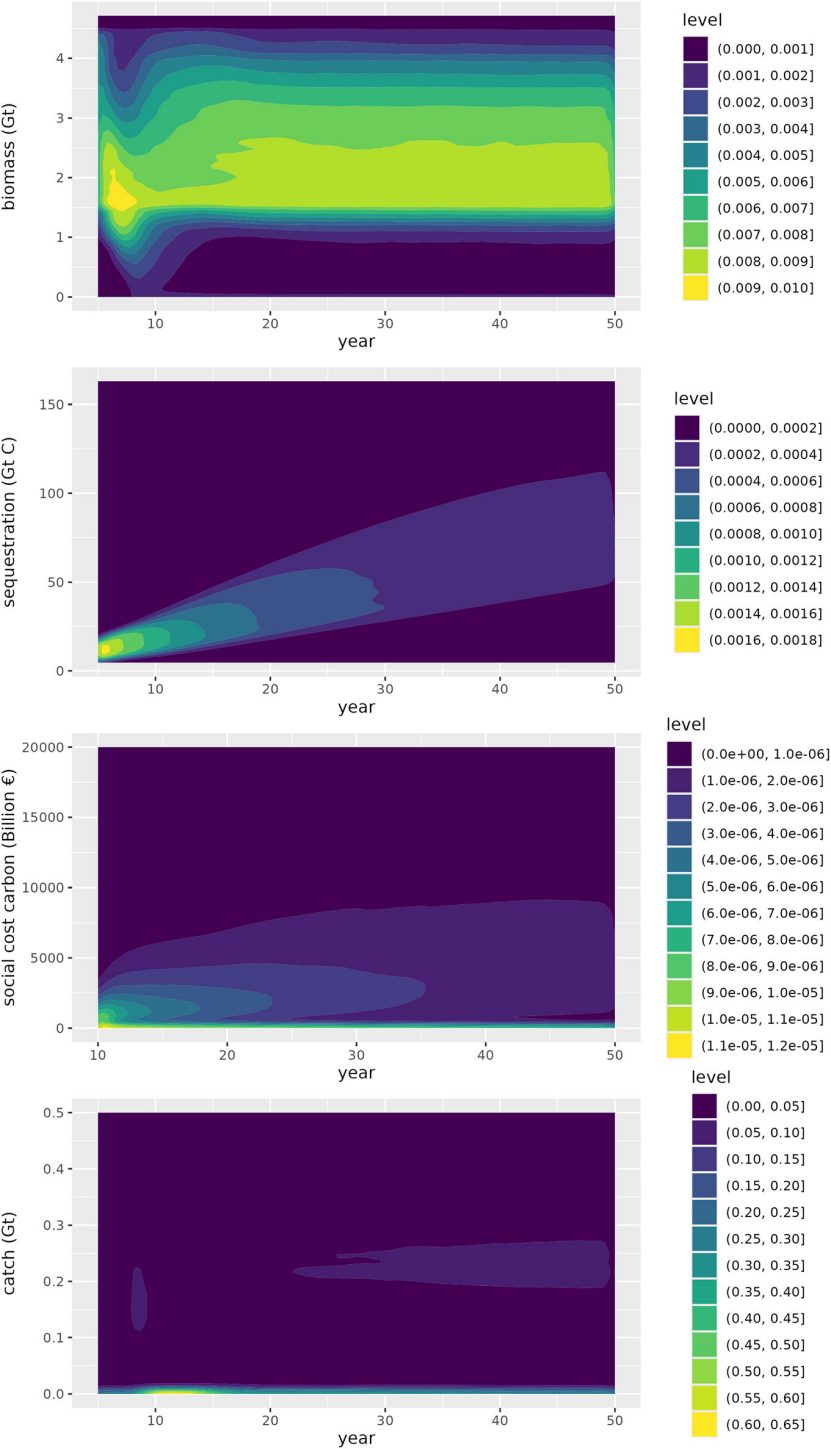
Density plots of 100 000 runs of the model with all uncertainties and its outcome for mesopelagic harvest, biomass and the valuation of carbon sequestration by mesopelagic fish. Level represents the density of observations. Workshop participant quotes on possible outcomes of fishing the mesopelagic associated with each of the modeled outcomes are depicted on the right side of the figure. Heavy tails were removed from the catch (15% of observations) and social cost of carbon (6% of observations) plots as those made it difficult to observe the distribution of most observations. Note also that plots start at year 5 of the simulation for all variables except social cost of carbon which start at year 10, due to the many zeros and low values at the start of the social cost of carbon, the density plot showed little of the actual distribution in later years

We found a wide range of possible outcomes for mesopelagic biomass and social cost of carbon, which is predominantly impacted by the uncertain amount of biomass in the mesopelagic zone (Fig. 2) and the uncertainties around the profitability of fishing. Overall, model runs suggest a median mesopelagic biomass of 2.5 Gt wet weight (Interquartile range = 1.4) (Fig. 2), which is somewhat lower than the median of 3 Gt (Interquartile range = 1.5) without harvest. Carbon sequestered is proportional to the mesopelagic biomass and is projected to be a median of 86Gt carbon (Interquartile range = 47 Gt), cumulative over the 50 years of the simulation (Fig. 2). We found a median of 0.22 Gt per year harvest, which is an extremely large amount of production, considering that it is more than three times the global total seafood production from wild capture, which was 0.09 Gt in 2020 (FAO 2022). Thus, the stakeholder perspective that this fishery could be meaningful for seafood supply is validated (Fig. 2, Appendix S1). The fishery was also profitable, with a yearly industry profit reaching a median of €39 206 million (Interquartile range = 85 404) by the end of the simulations. However, in 20% of runs, the profitability of the fishery was zero or below zero by the end of the simulation.

When we restricted the maximum yearly harvest to be around the size of the current global capture of forage fish (20 million tonnes), runs showed a median harvest of 20 million tonnes annually (and a mean of 16.7 million tonnes), at a cost to society of € 7 961 million a year (Interquartile range = 10 900 million) as measured by the social cost of carbon by the end of the simulation.

**Fig. 3:**
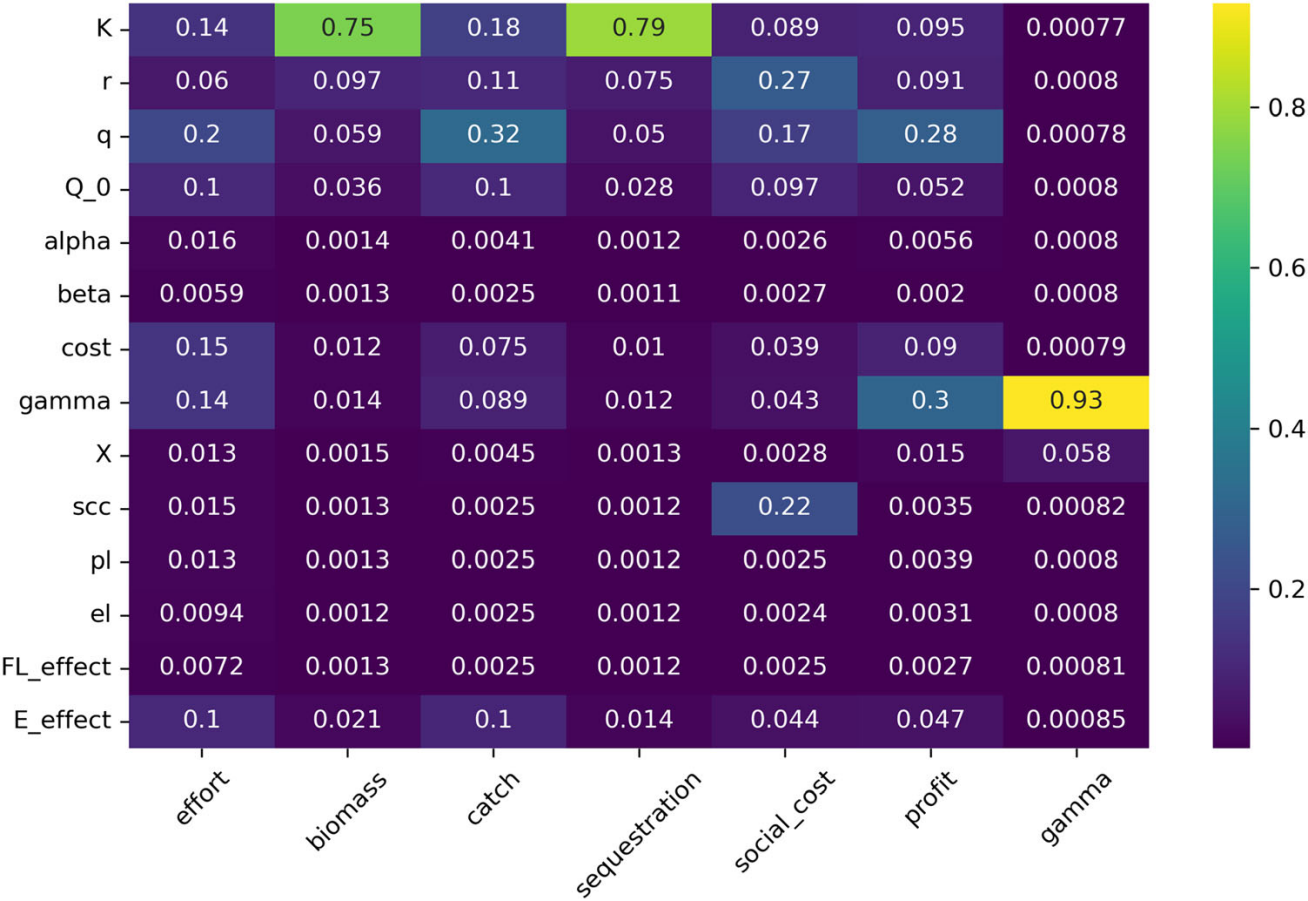
Feature scoring plot of 100 000 runs of the model with all social-ecological uncertainties and modeled outcomes for biomass, total sequestration, social cost of carbon, total catch, and total profits. Distributions show which uncertainties are driving most of the model behavior with regards to the modeled outcome, yellow meaning that the variable was driving much of the model behavior in many of the model runs. Model parameters are; K = carrying capacity, r = growth rate mesopelagic fish, q = catchability, Q_0 = advised quota, alpha = modifies effort with respect to the previous year's effort, beta = price flexibility, cost = fishing industry fishing cost per day, gamma = initial price of mesopelagic fish, X = demand multiplier, scc = the social cost of carbon, pl = profit level (%) at which fishing lobby effect starts to take effect, el = level of loss of carbon sequestration (%) at which environmental concern starts to take effect, FL_effect = fishing lobby effect, and E_effect = environmental protection effect. Outcome variables are; effort (in days), biomass (in Gt), catch (in Gt), sequestration (in Gt carbon), social cost (in Euros), profit (in Euros), modeled price of mesopelagic fish (gamma, in Euros). Remineralisation rates are excluded from the feature scoring plot as they have an extremely small impact due to the relatively short timescale of the model

Uncertainties in mesopelagic population characteristics driving these outcomes, and carrying capacity K is especially important for the modeled carbon sequestration (and social cost of carbon) of mesopelagic fish (Fig. 3). The uncertainty in the estimates of the social cost of carbon itself mainly impacts the evaluated social cost of carbon as do growth rate, quota, and catchability parameters. The faster mesopelagic fish grow, the smaller the impact of fishing on carbon sequestration and monetised climate damage. Catch, and profits are largely impacted by catchability of mesopelagic fish, its carrying capacity, the initial level of set quota, and environmental concern of the decision maker (Fig. 3). Initial demand is also an important variable mainly for the outcome of profitability of the fishery (Gamma, Fig. 3).

### Trade-offs between seafood supply and carbon sequestration

**Fig. 4:**
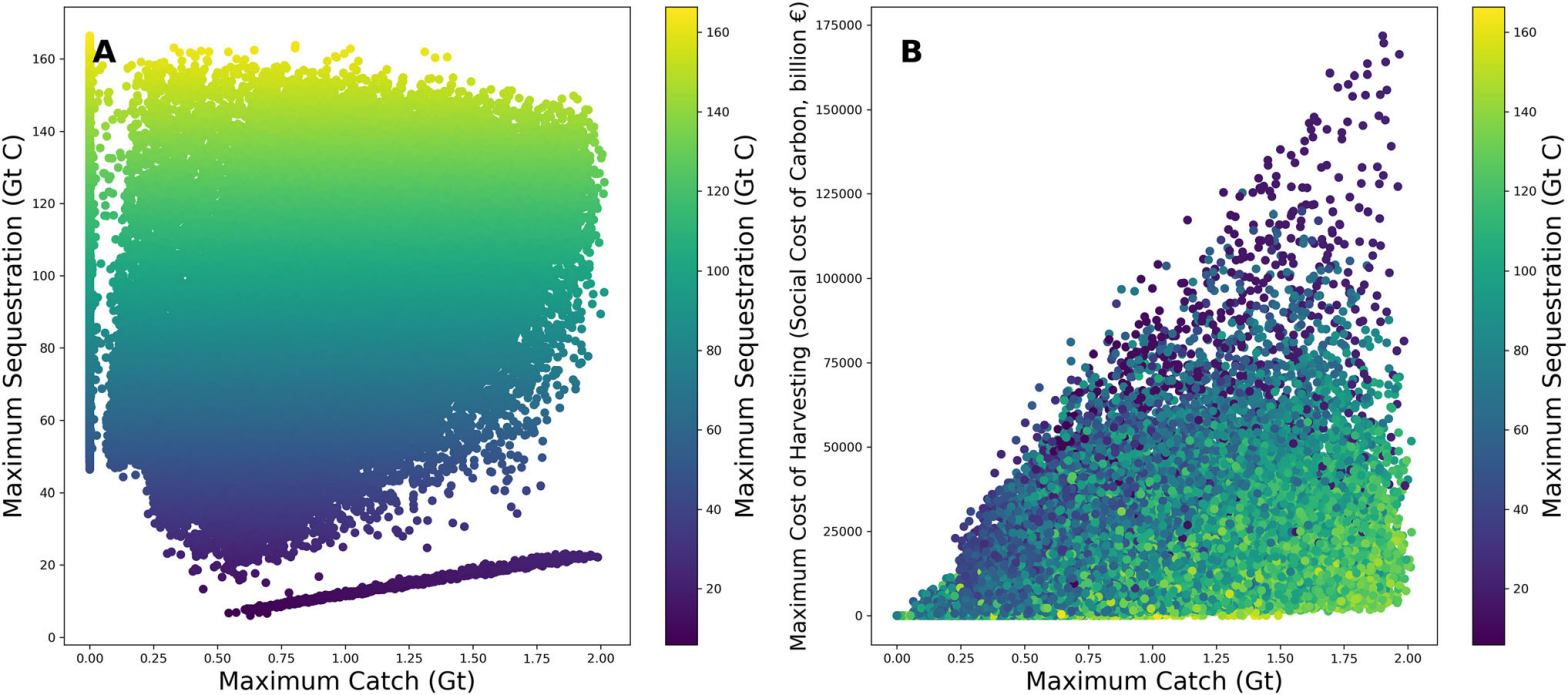
**A** Scatter of maximum carbon sequestration & seafood supply (color is valuation in social cost of carbon) (run is only including the economic and governance uncertainties), demonstrating the trade-off between carbon sequestration by mesopelagic fish and their contribution to seafood supply. **B** Scatter of maximum climate damage measured by the social cost of carbon of the fishery & seafood supply (color is in maximum sequestration)

The model results show a synergy between maximum catch levels and the maximum amount of carbon sequestration, mainly because both are higher when biomass is higher (Fig. 4A). However, there is a clear trade-off between carbon sequestration and catch, as is seen from high estimates for climate damage of the fishery, because of lost carbon sequestration ecosystem services with a decrease in the mesopelagic fish populations (Fig. 4B). The biggest loss of carbon sequestration and the highest cost in the social cost of carbon occurs in model runs with unsustainable exploitation, which also results in lower cumulative catches over the full timeline (Fig. 4A). The cost of the fishery to society, as measured by the social cost of carbon, is in the order of 7 trillion dollars (median, Interquartile range = 7.9, but with outliers, as seen in Fig. 4B).

### Governance analyses: Industry lobby versus environmental concern

We found that the modeled final year median biomass of mesopelagic fish with high levels of industry lobby and low level of environmental concern of the decision maker is 2.3 Gt (Interquartile range = 1.3). In comparison, the modeled median final year biomass of mesopelagic fish with low levels of industry lobby and high level of environmental concern of the decision maker is 2.6 Gt (Interquartile range = 1.4). The differences between the governance “scenarios” in terms of biomass and sequestration are small, mainly because of the large number of uncertainties impacting those outcomes, many of which are in the ecological system.

**Fig. 5:**
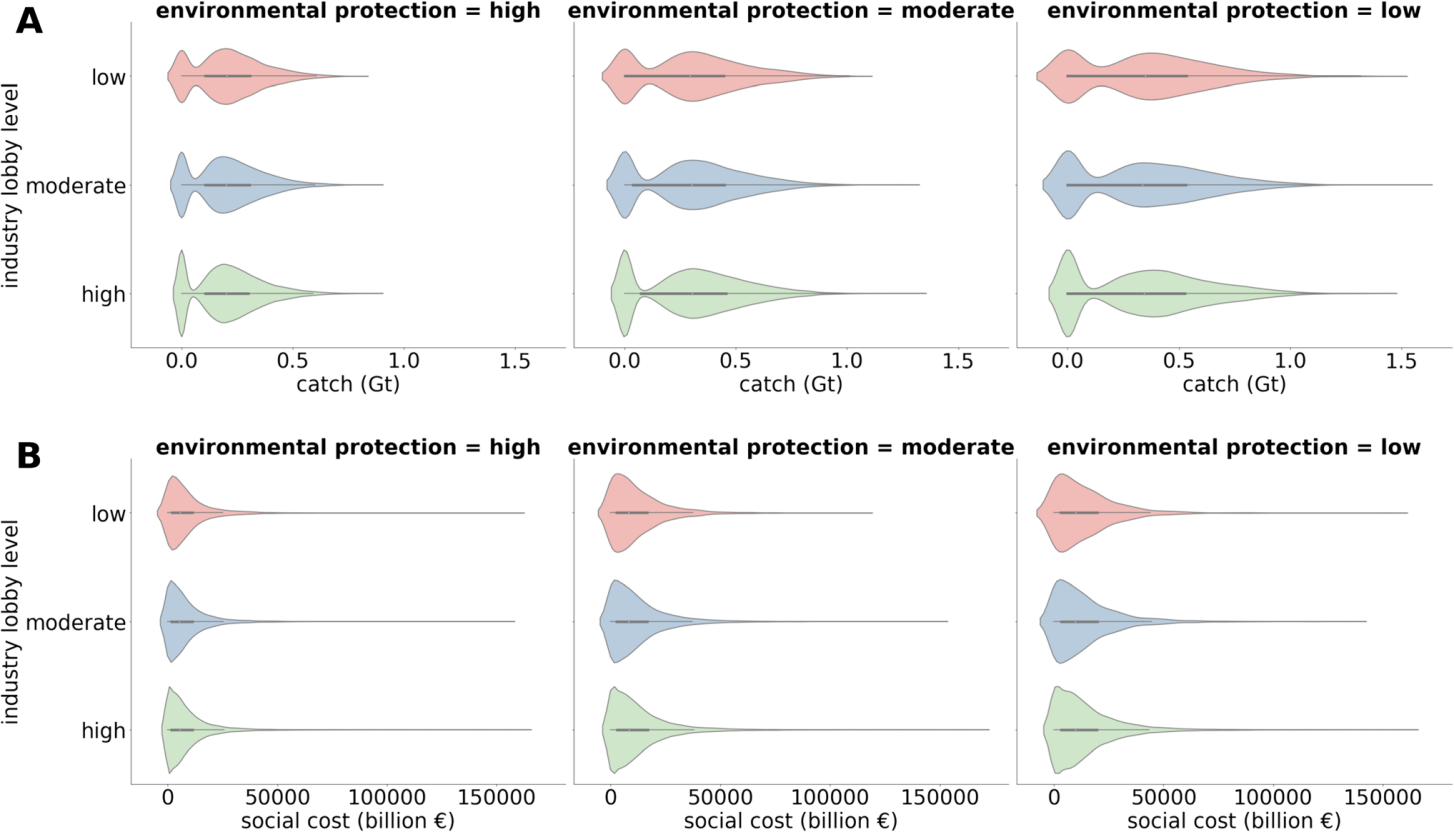
Violin plots of **A** final year catches, and **B** cumulative social cost of carbon of fishing in outcomes with high industry lobby versus high environmental concern of the decision maker, high environmental protection prevents high amounts of fishing that is very costly for society in terms of its cost in the social cost of carbon. Violin plots are density curves, i.e., where there are the most observations the outline and fill is the broadest. The long tails with few observations indicate a distribution with outliers. On the inside of the density curves, box plots are depicted showing medians as white dots and interquartile ranges with grey bars

Outcomes in levels of catch, and social cost of carbon from fishing are much more sensitive to the governance parameters (Fig. 5). Modeled median catch with high levels of industry lobby and low level of environmental concern was a median of 0.34 Gt in the final year. In comparison, catch in scenarios with low levels of industry lobby and high level of environmental concern had a median of 0.2 Gt (Fig. 5A). Modeled median valuation of the cost of loss carbon sequestering ecosystem service (based on the social cost of carbon) with high levels of industry lobby and low level of environmental concern of the decision maker is somewhat lower in the runs with high environmental protection (10 trillion Euros vs. 5.4 trillion Euros, Fig. 5B). The extreme outcomes are also more pronounced in the runs with a big impact of industry lobby and low level of environmental protection, with a maximum of 166 trillion Euros in terms of the social cost of carbon from fishing versus a maximum of 99 trillion Euros in a run with high environmental protection and rather low impact of industry lobby.

### Worst-case scenario discovery

**Fig. 6:**
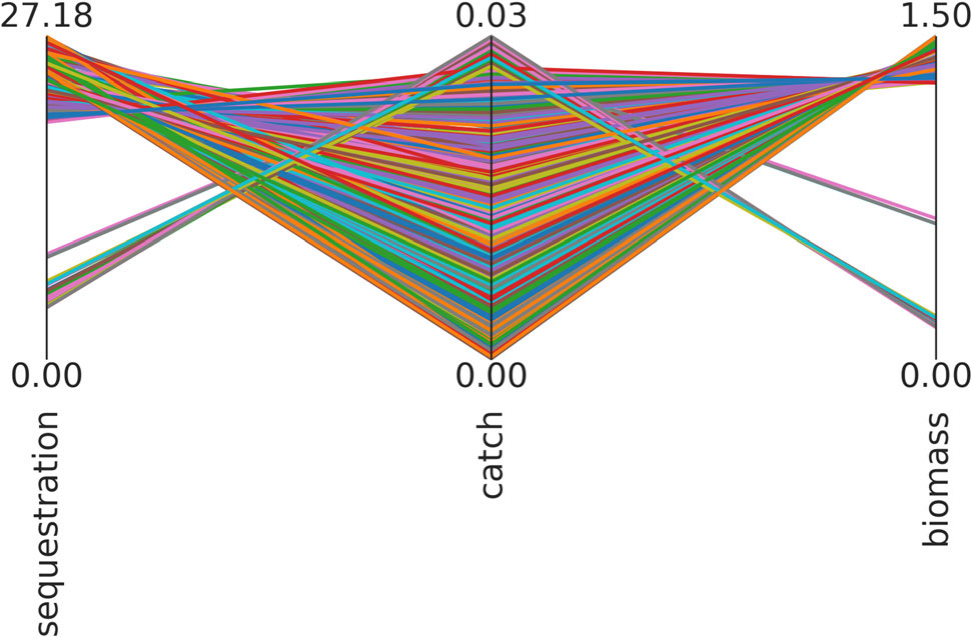
Worst-case scenario discovery, run over all uncertainties. The lines show combinations of (low) outcomes in the worst-case scenarios of the outcome variables mesopelagic fish biomass, total sequestration, and catches of mesopelagic fish (catch). Line colors differentiate individual runs, each line depicted is the outcome for the three outcomes of interest of a single model run where a worst-case scenario was the result

We found several ‘worst-case scenarios’, with rather low catches, and biomass being on the lower end of the uncertainty range (1.5 Gt) (Line plot in Fig. 6 depicts combined outcomes from each individual model run that resulted in a worst outcome). There is a small set of solutions where the catch is somewhat higher (~ 0.03 Gt), but biomass and sequestration are notably lower (min biomass = 0.15 Gt, min sequestration = 4.3 Gt). There tends to be a synergy between sequestration and biomass while, unsurprisingly, there is a trade-off between biomass and catch (Fig. 6).

## Discussion

We set out to model trade-offs between seafood supply and carbon sequestration in the mesopelagic zone under deep uncertainty and different scenarios for governance. There is a paucity of data on the role of mesopelagic fish in marine food webs (Dowd et al. 2022), their population sizes (Proud et al. 2019), and therefore also the role that these animals play in the biological pump (Anderson et al. 2019; Saba et al. 2021; Pinti et al. 2023). While a lot of new knowledge has been gained about the mesopelagic zone recently (Hidalgo and Browman 2019; Proud et al. 2019; Pinti et al. 2023; Schadeberg et al. 2023), major uncertainties remain (Drazen and Sutton 2017; Bisson et al. 2023). Because of these uncertainties, we used a stylized dynamic modeling approach, explicitly accounting for these uncertainties. The model addresses uncertainties such as: How will aquaculture demand develop over the next decades (Froehlich et al. 2018)?; Will mesopelagic fisheries ever become efficient and profitable (Fjeld et al., 2023) and parameter uncertainties (e.g., what is the population size of mesopelagic fish (Proud et al. 2019), what is the food conversion efficiency of mesopelagic fish (Anderson et al. 2019)). Exploring these uncertainties in our dynamic model, we found large differences in mesopelagic biomass and carbon sequestration due to large uncertainties in the food web and biological pump parameters. With increasing fishing, the projected costs in terms of the social cost of carbon (without including the greenhouse gas footprint), are generally high, approximating a median of 6 trillion dollars on average after 50 years of the simulation. This is roughly comparable to the entire carbon stock from mangroves in 1996 (Richards et al. 2020) evaluated using our baseline conversion rate for the social cost of carbon (Table 1). Of course, the amount of time that it would take to rebuild mesopelagic fishes (if they are not overexploited), versus mangrove carbon deposits would be on entirely different timescales.

### Major uncertainties with outsized impact on the system

The main driving uncertainty we identified for mesopelagic biomass and carbon sequestration was the carrying capacity of the mesopelagic fish population (Fig. 3). This stresses the need for further research into this ecosystem to restrain such uncertainties before starting large-scale exploitation, which could possibly jeopardize carbon sequestration potential (Anderson et al. 2019; Pinti et al. 2023). The market price of mesopelagic fish is one of the major drivers of catch in the model, in turn affecting both biomass and carbon sequestration. As such, without strong governance, global market value and demand for mesopelagic fish is the crucial force that will have the greatest impact on the ecosystem impacts of human activity in the mesopelagic zone, should catchability and technology for harvesting mesopelagic fish improve (Fig. 3).

### Outcomes for food supply, mesopelagic biomass, carbon sequestration and social cost of harvesting

There was a total yearly harvest of mesopelagic fish of 0.22 Gt on average across runs. This is a staggeringly large number: global marine capture fisheries landed 0.09 Gt in 2020 (FAO 2022). However, mesopelagic fish may mostly be destined for processing into fishmeal and fish oil, which has large losses along the supply chain (around 70% for fishmeal, Jackson, 2009). Thus, despite impressive harvest estimates, the practical implications for actual food production would be significantly lower. Another caveat worth noting is that such a high harvest rate would require upscaling fishing capacity (European capacity for harvesting mesopelagic fish has been estimated to be around 140,000–500,000 tons per year, Groeneveld et al. 2022), and large harvest levels in Areas Beyond National Jurisdiction, quite far removed from ports which would increase the costs of fishing.

Considering supply chain losses and conversion factors, a catch of around 0.22 Gt annually could result in around 60 million tonnes of fishmeal and 13 million tonnes of fish oil; if those would all be used to feed salmon, around 60 million tonnes of salmon could be produced which would be a significant contribution to micronutrients globally (Hicks et al. 2019). Since feed sources are increasingly land-based (Cottrell et al. 2021), an even higher amount of aquaculture fish could be produced, but with an increasing impact on land.

However, such a steep rise in demand for forage fish is not realistic considering modeled future demands for forage fish for aquaculture (Froehlich et al. 2018). When we restricted the maximum yearly harvest to be around the size of the current supply of forage fish (around 20 million tonnes) results showed a median of 20 million tonnes annually, which is still a very large contribution to global forage fish harvest. Thus, if harvests from the mesopelagic zone could match present-day forage fish catches, this would significantly contribute to the global sector. The cost to society, however, as measured by the social cost of carbon, was a median of € 7.961 billion a year in this set of runs. To put all these numbers into perspective, a recent estimate showed that ocean fisheries have released at least 0.73 billion metric tons of CO_2_ in the atmosphere since 1950, (including greenhouse gas emissions from fishing) which would amount to around 469 billion dollars cost to society (an average of roughly 7 billion a year), in the form of social cost of carbon (Mariani et al. 2020). However, Mariani et al. (2020) did not consider the carbon sequestering function of fishes (transport by e.g., fecal pellets) and weigh blue carbon only by biomass extracted from the ocean and thus not sequestred, which makes these numbers difficult to compare.

There was a decrease in the biomass of mesopelagic fish in most model runs. This was clearly attributable to fishing. Fishing biomass stabilized at around 2.5 Gt in model runs with fishing versus 3 Gt in model runs without fishing. The parameters driving these outcomes were growth rate and those associated with fishery profitability (cost and price) and governance variables, mainly environmental protection. Compared to fishing, uncertainties around the carrying capacity parameter (representing the actual current biomass of mesopelagic fish) had a much larger impact on carbon sequestration in the model runs. This stresses the importance of resolving major ecological uncertainties before starting large-scale exploitation (Anderson et al. 2019). Moreover, a nascent mesopelagic fishery would be a very fuel-intensive fishery (Vastenhoud et al. 2023); with governments around the world striving to lower dependence on fossil fuels, fishing mesopelagic fish, especially for reduction fisheries purposes, may not be in line with global goals of reducing carbon emissions. Other trade-offs will also need to be analyzed in a complete cost–benefit analysis, e.g., reduced food availability for predators of mesopelagic fish (Kourantidou and Jin 2022).

### Governance analyses

As expected, we found that the social cost of carbon related to harvesting mesopelagic fish is lower in scenarios with less industry lobby and more environmental protection. However, a more unexpected finding is that the more environmentally minded scenarios resulted in a decrease in extreme outcomes for the social cost of carbon of mesopelagic fishing. This was true across scenarios of high levels of industry lobby, due to the explicit feedback between the loss of carbon sequestration function and the policy makers' concern and intervention via quota (Fig. 5). These findings strengthen the case for ecosystem-based fisheries management to consider carbon sequestration an important ecosystem function of open ocean marine ecosystems (Elsler et al. 2022; Oostdijk et al. 2022). Applying carbon taxes, at a minimum, to the greenhouse emissions of the fishing fleet alone (Machado et al. 2021) could also be an effective way to minimize impacts from this potential fishery, given that it would likely have a high CO2 footprint, just from fuel use alone (Groeneveld et al. 2022; Vastenhoud et al. 2023).

### Limitations and future work

The analyses in this paper are subject to several limitations. First, there are major data limitations, and the quality of the available data mainly limits a model. For instance, there is a lack of data on the exact contributions of mesopelagic fish to the carbon pump, as carnivorous mesopelagic organisms are not sampled by conventional empirical methods to study the carbon pump (Boyd et al. 2019; Pinti et al. 2023). Furthermore, the amount of biomass of mesopelagic fish is highly uncertain (Anderson et al. 2019), so estimates will always differ by a large amount.

The model presented is highly stylized and highlights how different uncertainties impact estimates of the effects of harvesting mesopelagic fish. However, a benefit of this stylized model approach is that elaborate sensitivity analyses can be performed with limited computational resources. Because the model is highly stylized, complex interactions such as food web dynamics are not considered. There are uncertainties in the global food web with regard to the role of mesopelagic fish (Anderson et al. 2019; Dowd et al. 2022; Morzaria-Luna et al. 2022), and an interesting future research question would be how other populations will respond to harvesting (or otherwise impacting, e.g., by toxic plumes from deep-sea mining or oil spills) mesopelagic fish (Dowd et al. 2022; Morzaria-Luna et al. 2022). Food web interactions include prey populations such as vertically migrating zooplankton, which could theoretically grow larger with reduced predation from mesopelagic fish, replacing some of the carbon transport ecosystem function of mesopelagic fishes. Because of these potential interactions, losses of carbon sequestration due to the removal of mesopelagic fish, as presented in this paper, should be seen as illustrative, not exact. More elaborate ecosystem models (e.g., FEISTY, Petrik et al. 2019; van Denderen et al. 2021) could be used to more specifically investigate the impact the removal of mesopelagic fish might have on other populations and carbon cycling processes. Such models (that are often individual-based) may also integrate uncertainties regarding bioenergetics and can study their impact on carbon cycling and sequestration (McMonagle et al. 2023). These uncertainties were not integrated in our present study (as these uncertainties relate in complex ways to parameters in the surplus production model we based our analysis on), but can have a large impact on projected quantities of carbon cycled and sequestred, i.e., sensitivity analysis revealed a sixfold difference in carbon sequestration within plausible bioenergetics parameters (McMonagle et al. 2023).

Again other models may be more suitable to estimate the viability and potential scale of mesopelagic fishery, notably models with a regional focus, spatial dynamics, and highly resolved technological detail of the fishing fleet can expose new limitations to this nascent fishery. For instance a recent study found that current pelagic vessels in Denmark that may be used to exploit mesopelagic fish indicated fuel tank capacity as a limiting factor due to the sheer distance of the fishing grounds (Vastenhoud et al. 2023).

Lastly, stakeholders for the participatory modeling sessions were mainly from companies and institutions in EU countries, where currently much of the interest in developing mesopelagic fisheries is concentrated (Kraan et al. 2022). However, since the stylized model is at a global level, other dynamics or extreme outcome scenarios might have been unveiled if a more diverse stakeholder group was consulted.

## Conclusion

Using a stylized modeling approach we synthesize information on the largest ecological, economic, and social uncertainties regarding the development of potential mesopelagic fisheries. There is a trade-off between carbon sequestering services of the mesopelagic zone and seafood supply. The magnitude of this trade-off is uncertain but is likely to be proportionate to the quantities of mesopelagic fish extraction. The quality of the data about the population size of mesopelagic fish, as well as the precise mechanisms of the carbon cycle, are major limitations to the ability of models to inform policy about these trade-offs. Our social-ecological modeling approach showed a potentially profitable fishery with a high CO_2_ footprint under most assumptions. Governance scenarios that prioritized prevention of further loss of carbon function as opposed to industry lobby showed lower costs to society in the form of the social cost of carbon. A precautionary approach to the management of mesopelagic fish is needed to preserve their important role in carbon sequestering.

## Supplementary Information

Below is the link to the electronic supplementary material.

Supplementary file1 (PDF 1285 KB)

## Data Availability

The only original data in the article is stakeholder data, more of these findings are presented in the report of Kraan et al. (2022). All other data and sources are summarized in parameter tables in manuscript or appendices.
