# Supplementary material for: Modeling fisheries and carbon sequestration ecosystem services under deep uncertainty in the ocean twilight zone
Source: Ambio. 2024 Aug 29;53(11):1632–48. doi: 10.1007/s13280-024-02044-1 (PMC11436683; doi:10.1007/s13280-024-02044-1)
Supplement: Supplementary file 1 — Supplementary file1 (PDF 1285 KB) [file 13280_2024_2044_MOESM1_ESM.pdf]

Modeling fisheries and carbon sequestration ecosystem services under deep uncertainty in the ocean twilight zone

Co-author list

Maartje Oostdijk [1,2,3]*, Laura G Elsler[4], Julie Van Deelen[5], Willem L. Auping[5], Jan Kwakkel[5], Amanda Schadeberg[6,7], Berthe Maria Johanna Vastenhoud[8], Claudiu Eduard Nedelciu[9], Fabio Berzaghi[10], Raul Prellezo[11], Mary S. Wisz[2]

*Corresponding author. Email: maartjeoostdijk@gmail.com, phone: 003547752065

1. Postdoctoral researcher, Agricultural University of Iceland, Árleynir 22, 112 Reykjavík, Iceland
2. Research associate (until December 2022). Ocean Sustainability, Governance and Management, World Maritime University, Fiskehamnsgatan 1, 211 18 Malmö, Sweden
3. Postdoctoral researcher. Science institute, University of Iceland, Saemundargata 2, 101 Reykjavik, Iceland.
4. Postdoctoral researcher. Harvard. T.H. Chan School of Public Health, Boston, MA 02115, United States.
5. Former Msc student. Policy Analysis Section, Department of Multi-Actor Systems, Faculty of Technology, Policy and Management, Jaffalaan 5, 2628 BX, Delft University of Technology, The Netherlands
6. Assistant professor. Policy Analysis Section, Department of Multi-Actor Systems, Faculty of Technology, Policy and Management, Jaffalaan 5, 2628 BX, Delft University of Technology, The Netherlands
7. Professor. Policy Analysis Section, Department of Multi-Actor Systems, Faculty of Technology, Policy and Management, Jaffalaan 5, 2628 BX, Delft University of Technology, The Netherlands
8. PhD student. Environmental Economics and Natural Resources Group, Wageningen University, Hollandseweg 1 6706 KN Wageningen, The Netherlands
9. PhD student. Environmental Policy Group, Wageningen University, Hollandseweg 1 6706 KN Wageningen, The Netherlands
10. PhD student. National Institute of Aquatic Resources, Technical University of Denmark, Kemitorvet 201, 2800 Kgs. Lyngby, Denmark
11. Postdoctoral researcher. Geography department, University of Bergen, Fosswinckels gate 6, Lauriz Meltzers hus 5007 Bergen, Norway
12. Research associate. Ocean Sustainability, Governance and Management, World Maritime University, Fiskehamnsgatan 1, 211 18 Malmö, Sweden
13. Principal Researcher. AZTI. Marine Research Unit. Txatxarramendi ugartea z/g, 48395 Txatxarramendi, Sukarrieta, Spain
14. Professor. Ocean Sustainability, Governance and Management, World Maritime University, Fiskehamnsgatan 1, 211 18 Malmö, Sweden

Author contributions:

Conceptualisation/study design: MO, LE, JD, WA, JK, MSW, Model design: MO, LE, JD, WA, Analyses: MO, LE, JK, Figures MO, Supervision: MSW, Writing first draft: MO, LE, All authors contributed to the writing

Acknowledgements

This work is delivered as part of the Horizon 2020 project MEESO Ecologically and Economically Sustainable Mesopelagic Fisheries (2019-2023), grant agreement No 817669. RP acknowledges funding from the European Union’s Horizon 2020 research and innovation program under grant agreements No. 817806 (SUMMER). We thank the stakeholders for participating in our interview campaign and the workshop. We also thank Marloes Kraan for her leading role in organizing the two stakeholder workshops and Rolf Groeneveld for supporting the organization of the stakeholder workshops, and comments on the paper.

Appendix S1. Participatory methods

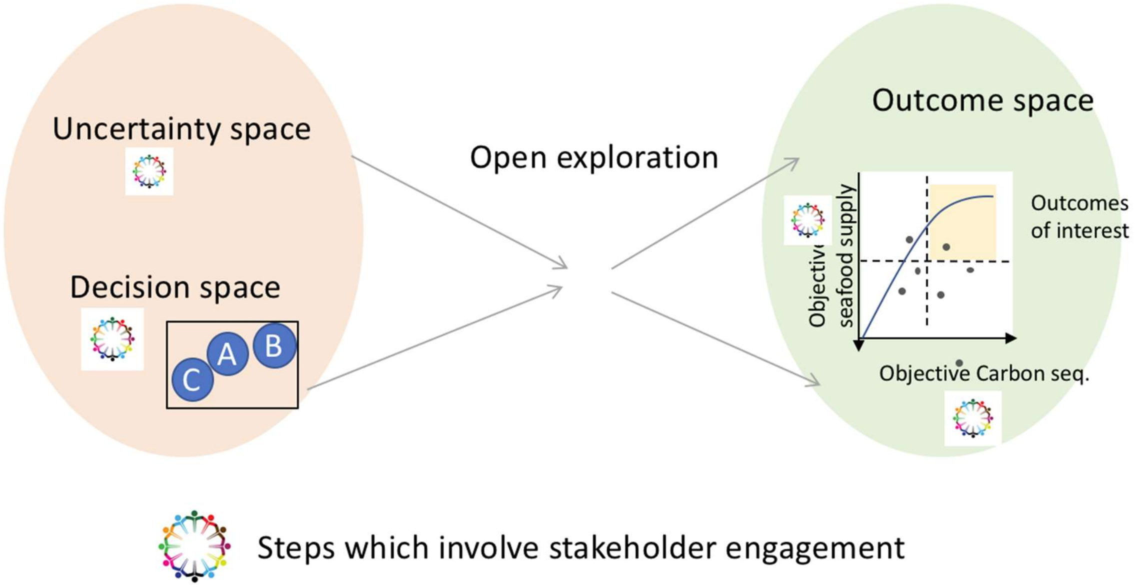

Figure S1: Adapted from Moallemi et al. (2020), use of stakeholder engagement in the participatory modeling process. Stakeholder involvement (by group model building) is used to determine (in part) the causal links in the model, this model informs the *uncertainty space* (all uncertainties of model parameters) on the top left of the diagram. Other parts of the *uncertainty space* are informed by an existing ecosystem model and equations and parameters derived from the literature. The *decision space* consists of predefined policy options (e.g. A) quota management, B) precautionary management, C) economic opportunities). The *decision space* is also informed by stakeholder participation. After an *open exploration* (running the models for many thousands of simulations), the *outcome space* is presented. This *outcome space* consists of objectives that are maximized/minimized. By using stakeholder involvement, we decide on which outcomes are key to maximize/minimize. A worst-case *scenario* is defined as the outcome with the most undesired (high or low, depending on the desired direction) outcomes across a set of outcome variables.

Supplementary Text.

To inform the core structure of the model (Figure 2) and the outcome spaces to strive for or avoid we used two main participatory methodologies 1) Participatory modeling (Zimmerman et al., 2016) using the mental modeler software (Gray et al., 2013) and 2) a previous interview campaign (Oostdijk et al., 2022). During the participatory modeling sessions (Kraan et al., 2022), held in Zoom September

2021, we had three breakout sessions each focusing on different possible outcomes for the mesopelagic zone and causal pathways associated with these outcomes. Break out groups were focused on outcomes for food security/seafood supply, consequences for the ecosystem/biodiversity and consequences for carbon sequestration. Breakout sessions had one note taker and one facilitator, the facilitator constructed the model in the mental modeler software in conversation with the group. Each group had 4-5 participants and the participants came from different backgrounds, Environmental Non-Governmental Organisations, a civil organization, fishing industry and academia. Outcomes of the mental models can be found in more detail in Appendix S2. A second workshop held in June 2022 was held to validate that the model was indeed addressing stakeholder concerns and we asked the stakeholders questions around which policies would most likely be implemented for (potential) mesopelagic fisheries.

Group model building on possible outcomes of fishing for the mesopelagic zone

Several negative possible impacts were identified by the workshop participants, including overfishing and biodiversity loss in mesopelagic fish populations (especially considering that there is so much unknown about the populations), loss of carbon sequestration function was also mentioned as a possible negative outcome of starting a fishery, as well as the greenhouse gas intensity of the fishery and the potentially large bycatch in the fishery. All possible positive outcomes of a mesopelagic fishery had to do with seafood supply and its contribution to food and nutrition security, given that the population is growing.

During one of the breakout groups several options for governance of the carbon sequestration functions of the vertically migrating mesopelagic fish were discussed. For instance, governance (of fishing) can be implemented via a moratorium (e.g. on the high seas) on fishing mesopelagic fish or through the setting of quota (either nationally, or through international agreements in international waters, see figure S1). The breakout group also discussed more complex methods linked to

governance, for instance the need of monitoring and enforcement to control illegal fishing and the valuation to the CO_2 sequestration of mesopelagic fish, for which knowledge on these functions is required. One of the participants suggested the use of national accounting of these carbon sequestration functions (of mesopelagic fish) and the impact on these functions in the case of mesopelagic fishing in nationally determined contributions for the Paris agreement (i.e. if a mesopelagic fishery would be developed the impact on the carbon pump would need to be reported on).

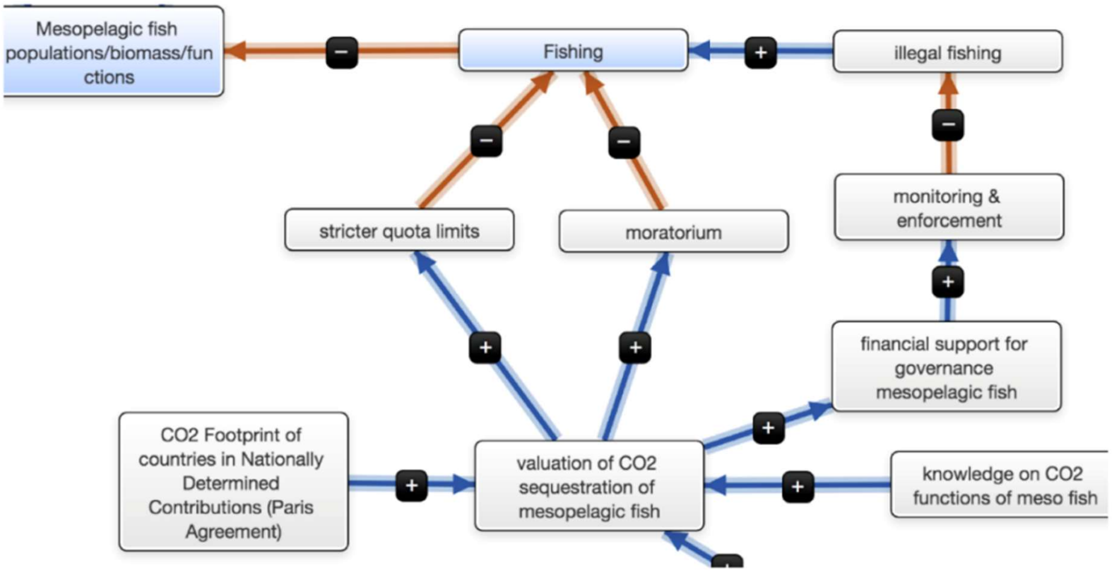

Figure S1: Carbon sequestration functions of the mesopelagic zone. What was imagined is if there is more biomass, more individuals fulfil the carbon sequestration function) this is incorporated in the model by adding function to biomass: mesopelagic fish populations/ biomass/functions (also reported in Kraan et al., 2022).

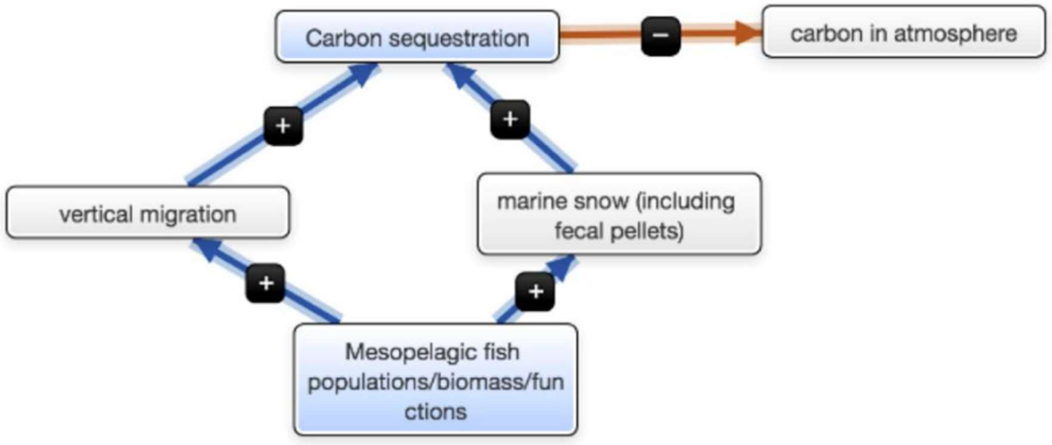

Figure S2: governance of the mesopelagic zone. Quota size will impact the amount of carbon sequestration increasing or decreasing (this causal connection is in the conceptual model) by impacting the biomass of mesopelagic fish populations.

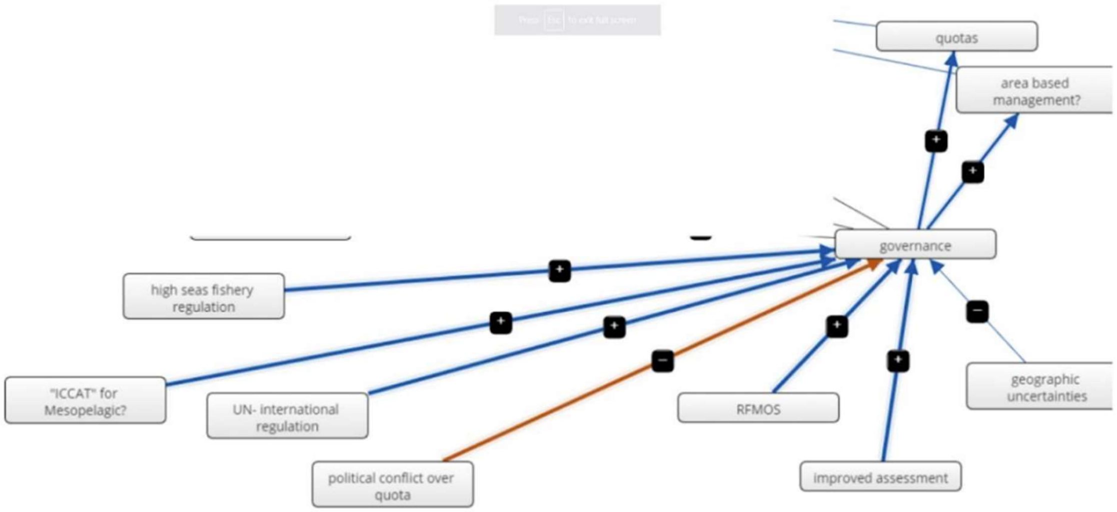

Figure S3: Governance pathways highlighted in the breakout room

In a second break out session, quotas and area-based management were the main management levers that we discussed that governance could use to influence fishing pressure. Governance of mesopelagic

fisheries could be positively supported by high seas fishery regulations, UN- international regulation, an "ICCAT" for the mesopelagic, improved assessment, work through RFMOs. Political conflict over quota and geographic uncertainties could challenge governance (Figure S3).

Appendix S2

Ecological Parameters

Estimates of mesopelagic biomass vary widely (1-16 Gt) but in most recent years biomass estimates seem to converge around 2Gt wet weight (Anderson et al., 2019; Pinti et al., 2023). Mesopelagic fish are fast growing species with high mortality (Anderson et al., 2019), are not very long lived (Caiger et al., 2021) and have high growth rates (Caiger et al., 2021).

Biological carbon pump parameters

Mesopelagic fish (and zooplankton) feed on zooplankton in the surface layer at night and migrate downwards to the mesopelagic zone during the day. This process is called Diel Vertical Migration (DVM). The consumed zooplankton contains carbon. Since the mesopelagic fish then respire, excrete, and defecate or die, the carbon ends up in the deep sea (Davison et al., 2013; Saba et al., 2021). Of these processes, respiration as a function of fish' metabolism is thought to be the greatest contributor to carbon export (Davison et al., 2013). As this happens at a greater depth and most importantly in or below the thermocline, the carbon is stored for a longer time than when this happens in surface waters (Boyd et al., 2019), therefore mesopelagic fish are the main contributor to carbon sequestered through the respiration pathway (around 70% of carbon sequestered this way, Pinti et al., 2023). Because vertical migrations enhance the velocity of vertical transport of carbon (versus the passive sinking of marine snow) they also reduce the reuptake of the carbon by microbes that would keep the carbon in the upper ocean layers, vertical migration patterns of fish therefore greatly determine how long carbon is stored (Saba et al., 2021; Pinti et al, 2023). Our model contains a highly simplified sequestration

process, where carbon injection via excretion, fecal pellets and mortality are based on the single species population growth model of mesopelagic fish and growth is dependent on the population size. There exists uncertainty about metabolic rates (Table 1), and there may be much more carbon transported via fecal matter than initially thought/modeled (Saba et al, 2021), however those uncertainties are not explicitly taken into consideration in our approach. These rates are based on daily metabolic rates assumptions regarding mesopelagic fish, that are not explicitly modeled in our analysis (and relate in a non-straightforward way to growth in a surplus production model, as r represents both recruitment and metabolic growth), we therefore chose the baseline scenario in Davison et al. (2013) for these estimates.

Economic Parameters

We assume that mesopelagic fishery from existing pelagic vessels will probably only happen if there is excess capacity for pelagic fishing (e.g. this could occur in a scenario of lower productivity due to climate change of other pelagic stocks). The current excess capacity of the pelagic fleet in “global North” nations is often rather close to zero (Paoletti et al., 2021).

However, some fleets with excess capacity have shown interest in the relatively shorter term such as the Irish and the Bask fleets (Groeneveld et al., 2022). However, in the future it is possible that specialized technology will be developed for harvesting mesopelagic species (Groeneveld et al., 2022) which could greatly expand fishing capacity and increase the cost of fishing (Paoletti et al., 2021). The costs of fishing mesopelagic fishing probably will be higher than for current pelagic stocks that are being fished and sales price is probably going to be lower (Paoletti et al., 2021), however the latter depends on if specialized products (e.g. nutraceuticals) will be developed (stJohn, 2016). Moreover, prices for aquaculture feed may increase with rising demand and decreasing supply of other forage fish (Froehlich et al., 2018), in Froehlich et al. (2018) several scenarios are described

regarding aquaculture production and use/demand for forage fish, based on these scenarios we use scenarios of up to 50% demand increase.

Governance parameters and structures

We assumed that quota setting is influenced by industrial lobbying, and that lobbying becomes stronger with increasing potential profits of mesopelagic fish (Edwards, 2000; Khalilian et al., 2010). We also assumed that quota setting (by e.g. the fishing ministry or agricultural ministry) can also be impacted by a decrease in carbon sequestration by fishes if this is believed to go against the public interest. Initial quota in the model is based on quota for blue whiting (Table 4) Blue whiting occupies the upper portions of the mesopelagic, is harvested primarily for fishmeal and fish oil production. A potential pathway for a lower quota setting after decrease in carbon sequestration ecosystem services could also be the implementation of taxation for carbon emissions, although such taxes would likely only apply to GHG emissions of the fleet, or the implementation of a market for blue carbon, including the carbon sequestering functions of mesopelagic fish. Such a carbon market was brought up during our interview campaign (Oostdijk et al., 2022) and one of the participatory modeling sessions (Kraan et al., 2022). Another pathway for such lowering of quota could be that the government takes a precautionary approach for the loss of carbon sequestration function (Table 2).

We ran the model for different levels of environmental concern of the (stylised) decision makers (e.g. the fishing ministries or agricultural ministries) and industry lobby/ industry/economic concern of the decision makers (Table 2, range for deep uncertainty analysis). We compared median and interquartile ranges for runs with high industry lobby and low environmental concern, low industry lobby and low environmental concern, high industry lobby and high environmental concern and low industry lobby and high environmental concern. This can be seen as the decision maker making a trade-off between various benefits and costs that are the result of a potential fishery, e.g. potential benefits include e.g.

contributions to economic expansion, job creation and food supply, while environmental impacts are the biggest potential cost to society.

Data and calculations for parameter estimation for economic module of the social-ecological model.

Value fishmeal and tonnes traded to estimate coefficient Beta (price flexibility).

- Fao trade data for several commodity groups, fishmeal from small pelagic marine fish, quantity and value obtained from: https://www.fao.org/faostat/en/ on February 2 2023
- Inflation data per country for IMF, obtained from : https://www.imf.org/external/datamapper/PCPIPCH@WEO/OEMDC on May 4th 2023
- Quantities lower than 100 tonnes per importing country per year were excluded as there is a lot of variability in price for small traded volumes

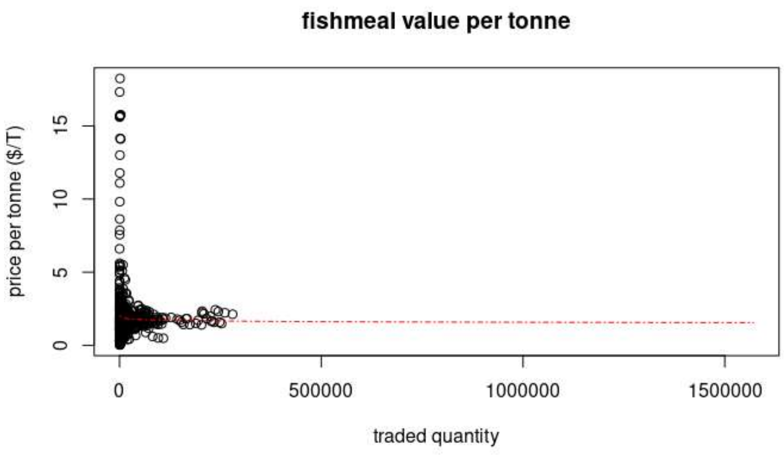

Figure S4. Price flexibility parameter b estimation (Venuzuela was removed from this dataset due to extreme inflation values)

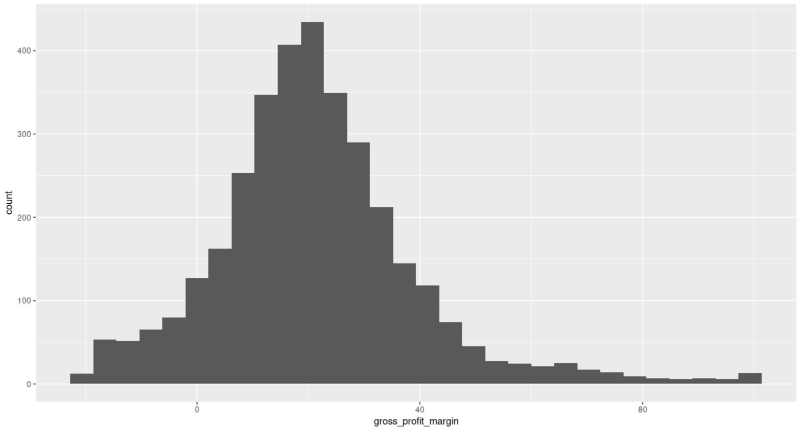

Figure S5. Histogram of gross profit margins of the EU fleet (2008-2018), excluding extreme loss making fleet segments (e.g. Malta) based on STECF data for the Anual Economic report

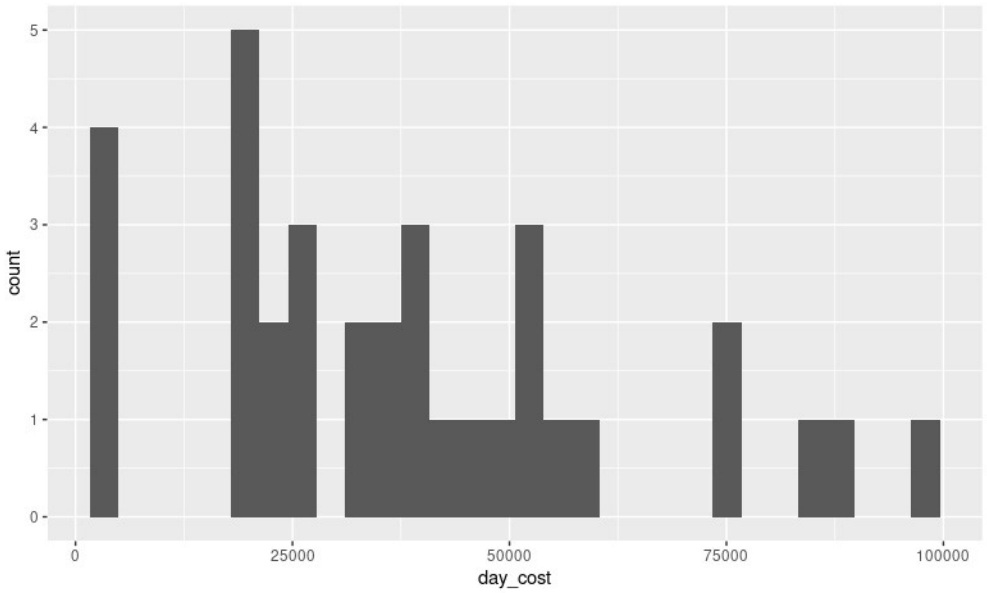

Figure S6. Histogram of costs per day for Pelagic Seines in EU waters 2010-2020, corrected for inflation in 2020 value, based on Annual Economic Report from STECFs. Link: https://op.europa.eu/en/publication-detail/-/publication/77fb8e7b-58a7-11ec-91ac-01aa75ed71a1/language-en

Supplementary references

Anderson, T.R., Martin, A.P., Lampitt, R.S., Trueman, C.N., Henson, S.A., Mayor, D.J., Link, J., 2019. Quantifying carbon fluxes from primary production to mesopelagic fish using a simple food web model. ICES J. Mar. Sci. 76, 690–701. doi:10.1093/icesjms/fsx234

Boyd, Philip W., Hervé Claustre, Marina Levy, David A. Siegel, and Thomas Weber. 2019. Multi-Faceted Particle Pumps Drive Carbon Sequestration in the Ocean. Nature 568, no. 7752. 327–35. https://doi.org/10.1038/s41586-019-1098-2.

Caiger, P.E., Lefebve, L.S., Llopiz, J.K., 2021. Growth and reproduction in mesopelagic fishes: A literature synthesis. ICES J. Mar. Sci. 78, 765–781. doi:10.1093/icesjms/fsaa247

Davison, P.C., Checkley, D.M., Koslow, J.A., Barlow, J., 2013. Carbon export mediated by mesopelagic fishes in the northeast Pacific Ocean. Prog. Oceanogr. 116, 14–30. doi:10.1016/j.pocean.2013.05.013

Dowd, S., Chapman, M., Koehn, L.E., Hoagland, P., 2022. The economic tradeoffs and ecological impacts associated with a potential mesopelagic fishery in the California Current. Ecol. Appl. 32, 1–17. doi:10.1002/eap.2578

Gray, S.A., Gray, S., Cox, L.J., Henly-Shepard, S., 2013. Mental Modeler: A fuzzy-logic cognitive mapping modeling tool for adaptive environmental management. Proc. Annu. Hawaii Int. Conf. Syst. Sci. 965–973. doi:10.1109/HICSS.2013.399

Groeneveld, R., Richter, A., Sen, S., 2022. Should we fish the mesopelagic? An economic analysis for four EU fishing fleets, Public Deliverable D6.3 for the EU MEESO project

Kraan, M., Oostdijk, M., Elsler, E., Schadeberg., A., Wisz, M., Groeneveld., R., (2022) Report: Report of the System and extreme outcomes workshop of the MEESO project held on 23 September 2021

Moallemi, Enayat A., Jan Kwakkel, Fjalar J. De Haan, and Brett A. Bryan. 2020. Exploratory Modeling for Analyzing Coupled Human-Natural Systems under Uncertainty. Global Environmental Change 65 102186.doi: 10.1016/j.gloenvcha.2020.102186.

Oostdijk, M., Elsler, L.G., Ramírez-Monsalve, P., Orach, K., Wisz, M.S., 2022. Governing Open Ocean and Fish Carbon: Perspectives and Opportunities. Front. Mar. Sci. 9, 1–15. doi:10.3389/fmars.2022.764609

Paoletti, S., Nielsen, J.R., Sparrevohn, C.R., Bastardie, F., Vastenhoud, B.M.J., 2021. Potential for Mesopelagic Fishery Compared to Economy and Fisheries Dynamics in Current Large Scale Danish Pelagic Fishery. Front. Mar. Sci. 8, 1–21. doi:10.3389/fmars.2021.720897

Pinti, J., DeVries, T., Norin, T., Serra-Pompei, C., Proud, R., Siegel, D.A., Kiørboe, T., Petrik, C.M., et al., 2023. Model estimates of metazoans' contributions to the biological carbon pump. Biogeosciences 20, 997–1009. doi:10.5194/bg-20-997-2023

St. John, M.A.S., Borja, A., Chust, G., Heath, M., Grigorov, I., Mariani, P., Martin, A.P., Santos, R.S., 2016. A dark hole in our understanding of marine ecosystems and their services: Perspectives from the mesopelagic community. Front. Mar. Sci. 3, 1–6. doi:10.3389/fmars.2016.00031

Zimmerman, L., Lounsbury, D.W., Rosen, C.S., Kimerling, R., Trafton, J.A., Lindley, S.E., 2016. Participatory System Dynamics Modeling: Increasing Stakeholder Engagement and Precision to

Improve Implementation Planning in Systems. Adm. Policy Ment. Heal. Ment. Heal. Serv. Res. 43, 834–849. doi:10.1007/s10488-016-0754-1
